# Reduced seed viability in exchange for transgenerational plant protection in an endophyte-symbiotic grass: does the defensive mutualism concept pass the fitness test?

**DOI:** 10.1093/aob/mcae133

**Published:** 2024-08-12

**Authors:** Benjamin Fuchs, Annelie Damerau, Baoru Yang, Anne Muola

**Affiliations:** Biodiversity Unit, University of Turku, Turku FI-20014, Finland; Food Sciences, Department of Life Technologies, University of Turku, Turku FI-20014, Finland; Food Sciences, Department of Life Technologies, University of Turku, Turku FI-20014, Finland; Biodiversity Unit, University of Turku, Turku FI-20014, Finland; Division of Biotechnology and Plant Health, Norwegian Institute of Bioeconomy Research, Ås, Norway

**Keywords:** Transgenerational plant resistance, grass endophytes, defensive mutualism, trophic interactions, insect pest control, growth–defence trade-off, induced alkaloid biosynthesis

## Abstract

**Background and Aims:**

*Epichloë* endophytes are vertically transmitted via grass seeds and chemically defend their hosts against herbivory. Endophyte-conferred plant defence via alkaloid biosynthesis might occur independently of costs for host plant growth. However, fitness consequences of endophyte-conferred defence and transgenerational effects on herbivore resistance of progeny plants are rarely studied. The aim of this study was to test whether severe defoliation in mother plants affects their seed production, seed germination rate and the endophyte-conferred resistance of progeny plants.

**Methods:**

In a field study, we tested the effects of defoliation and endophyte symbiosis (*Epichloë uncinata*) on host plant (*Festuca pratensis*) performance, loline alkaloid concentrations in leaves and seeds, seed biomass and seed germination rates. In a subsequent greenhouse study, we challenged the progeny of the plants from the field study to aphid herbivory and tested whether defoliation of mother plants affects endophyte-conferred resistance against aphids in progeny plants.

**Key Results:**

Defoliation of the mother plants resulted in a reduction of alkaloid concentrations in leaves and elevated the alkaloid concentrations in seeds when compared with non-defoliated endophyte-symbiotic plants. Viability and germination rate of seeds of defoliated endophyte-symbiotic plants were significantly lower compared with those of non-defoliated endophyte-symbiotic plants and endophyte-free (defoliated and non-defoliated) plants. During 6 weeks of growth, seedlings of defoliated endophyte-symbiotic mother plants had elevated alkaloid concentrations, which was negatively correlated with aphid performance.

**Conclusions:**

Endophyte-conferred investment in higher alkaloid levels in seeds, elicited by defoliation, provided protection from herbivores in progenies during the first weeks of plant establishment. Better protection of seeds via high alkaloid concentrations was negatively correlated with seed germination, indicating a trade-off between protection and viability.

## INTRODUCTION

Plants are constantly challenged by environmental stressors ranging from abiotic factors, such as drought, ozone or ultraviolet light, to biotic factors, such as competition, pathogen and herbivore attacks (Rejeb *et al.*, 2014; Suzuki *et al.*, 2014). To deal with herbivory, plants invest resources in defence-related metabolite biosynthesis at the cost of growth or reproduction (Koricheva *et al.*, 2004; Puijalon *et al.*, 2011; Huot *et al.*, 2014). In these situations, resources are allocated following the growth–defence trade-off concept (Huot *et al.*, 2014; Karasov *et al.*, 2017; Züst and Agrawal, 2017). Here, the trade-off between resource allocation to either growth or defence is balanced to achieve optimal fitness during herbivory (Huot *et al.*, 2014). Plant fitness is determined by survival and reproductive success, the latter of which is often neglected in studies on the growth–defence trade-off (Obeso, 2002; Monson *et al.*, 2022). Because growth determines the ability of plants to invest resources in reproductive structures, growth has been considered as a legitimate proxy for reproductive performance (Obeso, 2002). However, the simplicity of the concept of the growth–defence trade-off in plants is currently challenged, particularly when plants rely on microbial symbionts for defence (Grubb, 2016; Bastías *et al.*, 2021, 2022; Atala *et al.*, 2022).

Systemic endophytic *Epichloë* fungi are obligate endosymbionts of multiple cool-season grass species and are vertically transmitted via plant seeds (Schardl *et al.*, 2004). In moderate- to high-nutrient conditions, they improve plant growth, seed production and herbivore defence (Cheplick *et al.*, 1989). These fungal symbionts increase plant defence via the production of toxic alkaloids, which is commonly referred to as defensive mutualism (Clay, 1988, 2014; Faeth, 2002) and has a co-evolutionary history (Saikkonen *et al.*, 2004; Schardl *et al.*, 2004; Panaccione *et al.*, 2014). Alkaloid concentrations in grass–endophyte symbiosis vary across plant genotypes, endophyte strains, and biotic and abiotic conditions (Dirihan *et al.*, 2016; Fuchs *et al.*, 2020; von Cräutlein *et al.*, 2021; Laihonen *et al.*, 2022). Endophyte frequency and alkaloid concentrations are generally higher in areas with higher herbivore pressure, indicating that *Epichloë* endophytes provide a competitive advantage and evolutionary adaptation to herbivory (Saikkonen *et al.*, 2004, 2016; König *et al.*, 2018; Gundel *et al.*, 2020). Furthermore, feeding by herbivores induces alkaloid concentrations in endophyte-symbiotic plants without compromising plant growth, indicating a surpass of the growth–defence trade-off (Bultman *et al.*, 2004; Fuchs *et al.*, 2017*a*; Bastías *et al.*, 2021).

Two main factors, however, motivate a challenge of the assumption that endophyte-conferred defence can exist without a trade-off. First, in low-nutrient conditions, *Epichloë* endophytes reduce plant performance, which is caused by their high demand of nitrogen for alkaloid biosynthesis (Faeth and Sullivan, 2003). Second, endophyte-symbiotic seeds have a generally lower germination rate compared with endophyte-free plants, indicating that the endophyte impacts resource allocation of its host plant, with consequences to fitness (Faeth and Sullivan, 2003). Independent of herbivory, endophyte-symbiotic plants with high alkaloid concentrations show decreased growth, seed set and germination compared with endophyte-free plants, despite their elevated chlorophyl content (Faeth *et al.*, 2010; Rozpądek *et al.*, 2015), suggesting that alkaloid biosynthesis might trade off with host plant reproduction. Plants in general reduce the biosynthesis of defence compounds during seed development (Briggs and Schultz, 1990), indicating that resources are channelled towards seed development.

*Epichloë* endophyte survival and dispersal depend solely on host plant reproduction (Saikkonen *et al.*, 2004; Müller and Krauss, 2005). Furthermore, *Epichloë*-symbiotic plants produce seeds that contain high alkaloid concentrations for optimal herbivore protection (Müller and Krauss, 2005; Gundel *et al.*, 2012). Additionally, a recent study indicates an endophyte-mediated adaptive improvement in plant defence across plant generations, demonstrated by an accelerated inducibility of alkaloid concentrations (Bubica Bustos *et al.*, 2022). Under severe defoliation, endophyte-symbiotic plants are subjected to high stress, which might challenge the optimal resource allocation between the plant (growth) and the endophyte (defence). From an evolutionary point of view, the endophyte might invest all possible resources into protection of the next generation, whereas the host plant might primarily invest resources to achieve a high seed set as the best possible fitness outcome (Faeth, 2002; Koh and Hik, 2007; Friesen *et al.*, 2011). Here, we aim to elucidate the following: (1) whether defoliation throughout the growing season alters the viability (plant) and alkaloid concentrations (endophyte) of seeds (fitness–defence trade-off); and (2) whether defoliation of mother plants throughout the growing season alters the endophyte-conferred herbivore resistance of progeny plants.

We hypothesize that defoliation throughout the growing season: (1) induces the alkaloid concentrations and chlorophyl activity in leaves and seeds of endophyte-symbiotic plants; (2) causes a reduction in the seed set and germination rate in defoliated endophyte-symbiotic plants; and (3) increases the herbivore resistance of endophyte-symbiotic progeny plants.

## MATERIALS AND METHODS

### Study species

We used the perennial meadow fescue (*Festuca pratensis*) cultivar ‘Kasper’ as the study plant. It is often found to harbour a symbiotic fungal endophyte, *Epichloë uncinata* (Saari *et al.*, 2009). In this symbiosis, one genetic individual of *E. uncinata* forms a single haploid mycelium within the host plant and grows systemically and asymptomatically throughout its host, eventually into the inflorescence, from where it is distributed via the host plant seeds (Siegel *et al.*, 1987). The endophyte produces several pyrrolizidine alkaloids of the loline family in its host plant, with *N*-formylloline and *N*-acetylloline being the most abundant ones (Justus *et al.*, 1997). These alkaloids are produced exclusively in endophyte-symbiotic plants. Because the fungus is transmitted in a strictly vertical manner from the host grass into its progeny, the fitness of the fungus and of the host grass are tightly linked (Saikkonen *et al.*, 2004). We used seeds from endophyte-symbiotic (E^+^) and endophyte-free (E^−^) meadow fescues from the same cultivar grown for several generations within the study area as a seed stock.

### Study design of mother plants

The experiment was conducted in a common garden at the Botanical Garden of the University of Turku, Finland (60°26ʹN, 22°10.4ʹE). To consider possible environmental gradients within the experimental field, the plots (1 m^2^ in size) were arranged in a 6 × 6 grid consisting altogether of 36 plots. Plots were fertilized with quail manure before planting to ensure high soil nutrient conditions. At the end of September 2018, we planted two E^+^ and two E^−^*F. pratensis* seedlings per plot after germinating the seeds in the greenhouse for 18 days. The E^+^ and E^−^ individuals were planted crosswise in a double dichotomy setting within the plots. Plants were allowed to grow undisturbed during 2019 and 2020 to ensure their establishment on the field site. Above-ground biomass was harvested once per year, in August, after the seeds had developed. Because E^+^ plants have been shown to have a reduced alkaloid biosynthesis during their first years of growth (Fuchs *et al.*, 2017*b*), a 2-year period for establishment is justified. We conducted the defoliation treatment of the plants during the growing season of 2021 (May–July). Plants were not watered.

### Defoliation treatment

To test the effect of defoliation on the fitness of endophyte-free (E^−^) and endophyte-symbiotic (E^+^) mother plants and on alkaloid levels of endophyte-symbiotic (E^+^) mother plants, one E^+^ and one E^−^ plant per plot were randomly assigned to the defoliation treatment in spring 2021. This resulted in the following four treatments: endophyte-symbiotic *F. pratensis* with defoliation (E^+^C^+^); endophyte-symbiotic *F. pratensis* without defoliation (E^+^C^−^); endophyte-free *F. pratensis* with defoliation (E^−^C^+^); and endophyte-free *F. pratensis* without defoliation (E^−^C^−^). The aim of the defoliation treatment was to simulate heavy foliar grazing throughout the growing season. Defoliation treatment started at the beginning of the growing season, in May. Leaves were manually cut with sharp scissors to a remaining length between 3 and 5 cm per leaf. The emerging inflorescences were not cut. Defoliation was repeated every 5–7 days until mid-July, when the seeds were fully developed. Altogether, plants were defoliated ten times. Plants were monitored throughout the study period for pathogen infection and herbivore infestation, both of which remained negligible (data not shown).

### Photosynthesis measurements

To test whether defoliation and endophyte symbiosis affected photosynthetic activity of mother plants, we measured their foliar chlorophyll content with a hand-held optical chlorophyll meter (Soil Plant Analysis Development SPAD-502 Plus, Konica Minolta, Japan). Measurements were taken at three time points during the defoliation treatment: once at the beginning of the study, before the first defoliation in May, then once in June and once in July before defoliation. Measurements with the SPAD-502 meter produce relative values that are proportional to the amount of chlorophyll in the leaf. For the present study, we were interested only in the relative differences between the treatments and did not convert the values into absolute units. We abbreviate the presented unit as the SPAD value.

### Plant sampling

Plant biomass was harvested on 15 July 2021, when the seeds were fully developed but still largely attached to the inflorescences; this was carried out by manually cutting the entire above-ground plant biomass with a hand sickle. Above-ground biomass was placed in paper bags and transported to the laboratory. In the laboratory, the number of inflorescences was counted, and they were weighed. After that the seeds were stored in paper bags at 4 °C until they were processed for seed staining, alkaloid extraction and germination assay (see below). A subset of 100 randomly chosen seeds per mother plant was weighed in bulk on a fine scale (Mettler Toledo AX 204) to determine the effect of treatment on seed weight. The remaining above-ground biomass was oven dried (72 h at 65 °C) and weighed. Before the first defoliation event in May and at harvest in July, three randomly chosen tillers of E^+^C^+^ and E^+^C^−^ plants were collected and flash frozen in liquid nitrogen before lyophilization and homogenization for alkaloid analyses. An ammount of 50 mg of powdered leaf material was transferred to a 2 mL Eppendorf reaction tube for alkaloid extraction.

### Seed staining

Ten randomly chosen seeds per mother plant were analysed microscopically for the presence of endophyte hyphae. The seeds were placed individually in 2 mL reaction tubes (Eppendorf), and a 1 mL 95:5 water:ethanol (0.625 m NaOH) solution was added. The seeds were kept in the solution for 12 h in total darkness to soften cell walls, after which they were treated with Aniline Blue to visualize fungal hyphae. Fungal presence in the seeds was inspected via microscopy. The hyphae of the endophytic fungus can be detected between aleurone cells in the seed of the E^+^ plants, and we followed a published protocol for endophyte visualization and quantification (Belanger, 1996).

### Study design of progeny plants

In March 2022, 20 randomly chosen seeds from each mother plant were sown into seed trays with soil (Kekkilä Garden Viherkasvimulta). Seeds were placed on the soil surface before addition of a thin layer (2–3 mm) of fine soil. The trays were placed in a temperature- and light-controlled climate cabinet with a 16 h–8 h light–dark cycle and at corresponding 18–16 °C temperature to monitor the seed germination by daily observation for a period of 2 weeks. Approximately 2 weeks after germination, alkaloid concentrations were determined from the above-ground parts of ten randomly selected progeny plants per mother plant treatment (i.e. E^−^C^−^, E^−^C^+^, E^+^C^−^ and E^+^C^+^; 40 seedlings in total; for details see ‘Loline extraction and quantification’). Additionally, ten randomly selected progeny plants per mother plant treatment were transferred individually into 1.5 L pots containing soil (Kekkilä Garden Viherkasvimulta). The pots were placed in perforated cages (Bug Dorm 4E3074) in a greenhouse at 16 h–8 h light–dark cycle and at 18–16 °C temperature. Each caged plant received ten wingless adult *Rhopalosiphum padi* aphids, which had reached the last instar 1–3 days before. *Rhopalosiphum padi* were purchased from Katz Biotech AG and reared on oat seedlings in climatic conditions in line with the experimental settings in the greenhouse. Aphid numbers (on both E^+^ and E^−^ plants) and alkaloid concentrations (only on E^+^ plants) were monitored every 2 weeks for a period of 6 weeks. After 6 weeks of aphid infestation, a final aphid count was conducted before aphids were removed from the plants with a fine brush. All E^+^ plants were grown for an additional 12 weeks without aphids, and alkaloid levels were monitored every fourth week. Approximately 50 mg leaf material was collected for alkaloid analyses at each sampling time.

### Seed viability

To test whether the observed germination rate was correlated with seed viability and exclude the potential effect of seed dormancy on the results of germination assays, we performed a tetrazolium test on ten randomly selected seeds per mother plant, following a published protocol (Soares *et al.*, 2016). The tetrazolium test assesses seed viability based on a staining method. In brief, seeds were soaked in tap water for 4 h at 30 °C, then cut laterally, slightly above the embryo, and soaked in 1% tetrazolium solution overnight. The solution was removed, and 100 µL of 85% lactic acid was added to each ten seeds and incubated for 1 h at 30 °C to facilitate the visual identification of the embryo. A seed was considered viable when the entire embryo was evenly stained red (Soares *et al.*, 2016).

### Loline extraction and quantification

**Fig. 1.**
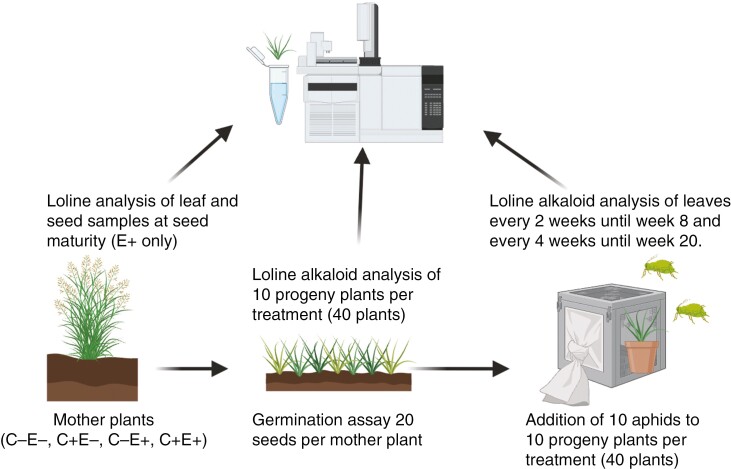
Experimental set-up and sampling scheme of loline alkaloid analysis of endophyte-symbiotic (E^+^) mother plants and their progeny. Graphical illustration created with biorender.com.

Lolines were extracted from samples collected from the following: (1) mother plant leaves once before the first defoliation in May 2021 and at seed maturity before final biomass harvest in July 2021; (2) seeds of the mother plants collected in July 2021; and (3) leaf material of progeny plants at seven time points from March to May 2022 (prior, during and after progeny plants were exposed to aphid herbivory) (Fig. 1). Lolines were extracted from ~50 mg of lyophilized and homogenized plant/seed material. Loline alkaloid concentrations were determined via gas chromatography coupled with mass spectrometry (GC-MS) modified after a published protocol (Pan *et al.*, 2014; Fuchs *et al.*, 2023). The plant material was transferred into 2 mL reaction tubes (Eppendorf) and mixed with NaOH (100 µL, 1 m). Afterwards, 1000 µL of chloroform was added, spiked with 50 µg mL^−1^ internal standard compound quinoline (Sigma Aldrich) and shaken at room temperature at a rate of 300 min^−1^ for 12 h (VWR incubating mini shaker). The extracts were centrifuged at 14 000*g* for 15 min (Eppendorf centrifuge 5415D), and the supernatant was passed through a PTFE syringe filter (0.45 µm) into 1.5 mL glass vials (Thermo Fischer). Vials were stored at −20 °C until injection of 1 µL into the GC-MS system. Detailed GC-MS parameters can be found in the paper by Fuchs *et al.* (2023). Concentrations of *N*-formylloline and *N*-aetylloline were combined because of their similarity in effectiveness against herbivores.

### Statistics

The effect of defoliation and endophyte status on biomass, number of inflorescences, seed biomass and SPAD value of the mother plants were analysed with a generalized linear mixed model, followed by Tukey’s *post hoc* test. For SPAD value analyses, ‘time’ was added as random factor into the model to account for repeated measurements taken from the same plants (three times) during the period of the defoliation experiment. The plot was included in the mixed model as a random factor. Pearson correlation was used to test the association between seed biomass and plant biomass, and between seed biomass and germination rate. The effects of defoliation treatment on loline concentrations in the leaves and seeds of mother plants were analysed with Student’s *t*-test. The effect of defoliation and endophyte status on seed germination was analysed using a general linear model and Tukey’s *post hoc* test. The effect of defoliation on loline concentrations in progeny plants and on aphid numbers on progeny plants was analysed with a generalized linear mixed model including plant individual as a random factor (accounting for repeated measurement) followed by Tukey’s *post hoc* test. Graphical illustration was done with the package ‘ggplot2’ in the software R (v.4.2.1).

## RESULTS

### Performance in mother plants

**Fig. 2.**
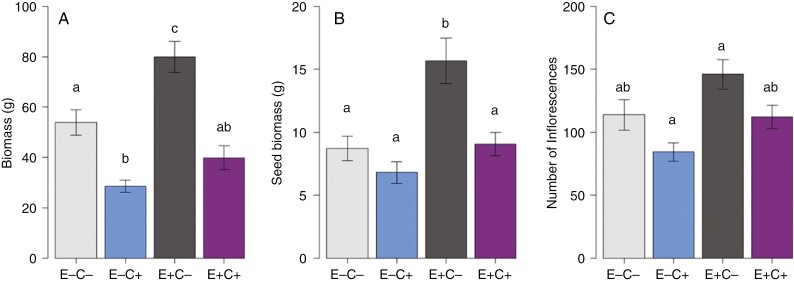
Plant biomass (in grams) (A), seed biomass (in grams) (B) and number of inflorescences (C) of endophyte-free (E^−^) and endophyte-symbiotic (E^+^) defoliated (C^+^) and non-defoliated (C^−^) meadow fescues at the end of the field study in July 2021. Lowercase letters indicate significant differences between treatments (Tukey’s *post hoc* test). Values are presented as the mean ± s.e.; *n* = 36. For statistical details, see Table 1.

Defoliation caused a reduction in leaf biomass (excluding seeds) of the mother plants (Fig. 2A; Table 1). The highest biomass was observed in endophyte-symbiotic non-defoliated (E^+^C^−^) plants. Plants with higher biomass produced more seeds across the treatments (*t* = 10.52, *P* < 0.001, *R*^2^ = 0.74). The endophyte-symbiotic non-defoliated (E^+^C^−^) plants showed the highest seed biomass and number of inflorescences (Fig. 2B, C; Table 1). Defoliation caused only a marginal loss in seed biomass of E^−^ plants compared with E^+^ plants, where defoliation significantly reduced seed biomass (Fig. 2B; Table 1). Seed weight of a subset of 100 seeds per plant did not differ between the treatments (ANOVA: *F* = 0.84, *P* = 0.48). Treatment did not affect SPAD values at any time point (Table 1).

**Table 1.** The results of generalized linear models testing the effect of treatment (endophyte-symbiosis (E) and defoliation (C); E^−^C^−^, E^−^C^+^, E^+^C^−^ and E^+^C^+^) on leaf chlorophyll content (SPAD) at three time points during summer 2021, plant biomass, seed biomass and number of inflorescences of meadow fescue ‘mother plants’ (mean ± s.e.). In progeny plants, the treatment refers to the origin of seeds produced by the mother plant with the respective treatment history. Significant differences between treatments tested with Tukey’s *post hoc* test are indicated by superscript letters next to the mean values. Week refers to the time since plant germination for progeny plants. Aphid treatment started at week 2. For graphical visualizations, see Fig. 5B.

Response variable	Explanatory variable	ANOVA			E^−^C^−^	E^−^C^+^	E^+^C^−^	E^+^C^+^
		D.f., d.d.f.	*F*	*P*-value	Mean ± s.e.	Mean ± s.e.	Mean ± s.e.	Mean ± s.e.
**Mother plants**								
SPAD May	Treatment	3,88	2.62	0.06	28.5 ± 1.0	27.1 ± 0.9	30.2 ± 1.5	31.0 ± 0.8
SPAD June	Treatment	3,88	1.15	0.33	30.4 ± 0.5	29.8 ± 0.8	30.6 ± 1.2	32.1 ± 1.0
SPAD July	Treatment	3,88	2.48	0.07	24.2 ± 2.1	25.5 ± 1.04	26.7 ± 0.7	27.8 ± 1.9
Biomass	Treatment	3,88	21.11	<0.01	53.9 ± 5.1^a^	28.6 ± 2.5^b^	80.0 ± 6.1^c^	39.9 ± 4.8^ab^
Seed biomass	Treatment	3,88	10.24	<0.01	8.7 ± 0.9^a^	6.81 ± 0.9^a^	15.7 ± 1.8^b^	9.06 ± 0.9^a^
Inflorescences	Treatment	3,88	6.08	<0.01	113.9 ± 12.1^ab^	84.4 ± 7.2^a^	146.0 ± 11.5^b^	112.3 ± 9.2^ab^
Germination	Treatment	3,88	18.96	<0.01	17.7 ± 0.4^a^	17.4 ± 0.5^ab^	15.5 ± 0.6^b^	12.0 ± 0.8^c^
**Progeny plants**								
Aphids week 4	Treatment	3,36	8.50	<0.01	38.8 ± 2.4^a^	39.8 ± 1.5^a^	35.8 ± 1.6^a^	28.4 ± 1.4^b^
Aphids week 6	Treatment	3,36	69.25	<0.01	110.5 ± 4.1^a^	112.3 ± 3.6^a^	84.2 ± 3.9^b^	51.6 ± 1.4^cc^
Aphids week 8	Treatment	3,36	151.0	<0.01	200.1 ± 4.7^a^	196.1 ± 5.5^a^	124.6 ± 6.0^b^	78.8 ± 1.8^c^

Abbreviations: d.f., d.d.f., numerator and denominator degrees of freedom rounded to the nearest integer.

### Endophyte frequency and alkaloid concentration in mother plants

**Fig. 3.**
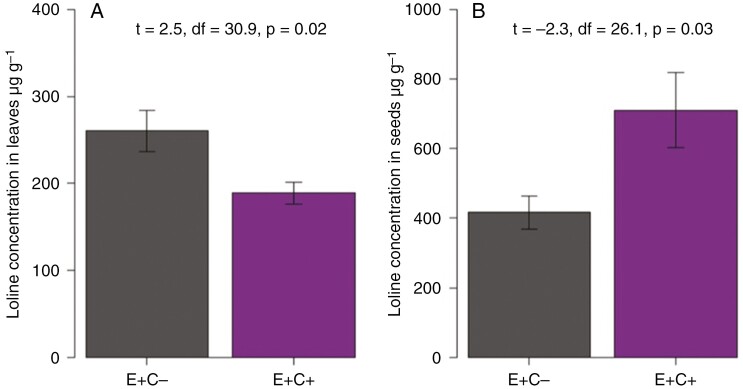
Loline alkaloid concentrations in leaves (A) and seeds (B) of endophyte-symbiotic (E^+^) defoliated (C^+^) and non-defoliated (C^−^) meadow fescue mother plants in the field study. The values are presented as the mean ± s.e., in micrograms per gram dry weight; *n* = 36.

Microscopic analysis of the seed stock used for this experiment revealed an average of 94% of endophyte infection of E^+^ seeds, and no difference in the amount of fungal mycelium per seed was detected before the start of the experiment. Loline alkaloid concentrations did not differ between the E^+^ plants assigned to different treatments at the start of the defoliation treatment in May 2021 [Student’s *t*-test: *t* = −0.37, *P* = 0.714, E^+^C^−^ mean ± s.e. = 297.8 ± 26.4 µg g^−1^ dry weight (DW); E^+^C^+^ mean ± s.e. = 316.1 ± 29.8 µg g^−1^ DW]. At the end of the experiment, loline alkaloid concentrations in the leaves of E^+^C^+^ plants were lower than those of E^+^C^−^ plants (Fig. 3A). In contrast, the alkaloid concentrations of seeds produced by the E^+^C^+^ plants were higher than those of E^+^C^−^ plants (Fig. 3B).

### Germination rate and viability of seeds

**Fig. 4.**
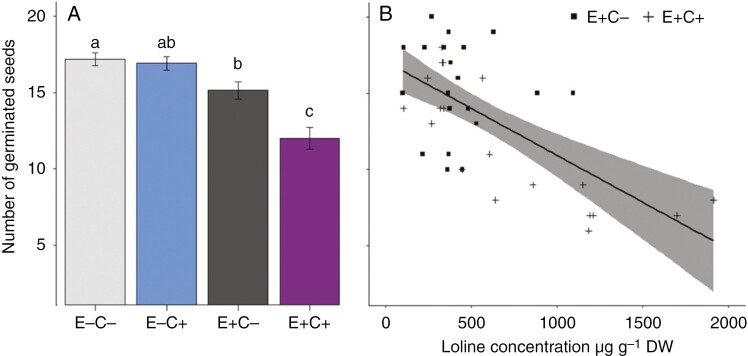
(A) Number of germinated seeds per 20 seeds produced by endophyte-symbiotic (E^+^) and endophyte-free (E^−^) defoliated (C^+^) and non-defoliated (C^−^) meadow fescues. Values are presented as the mean ± s.e. (B) Seed germination was negatively correlated with loline concentration in seeds in E^+^ plants, shown by the regression line with 95% confidence interval; *n* = 40.

Ninety-four per cent of seeds produced by E^−^C^−^ plants and 89% of those produced by E^−^C^+^ plants germinated. Seeds of E^+^C^+^ plants germinated at a rate of 61%, whereas seeds of E^+^C^−^ plants germinated at a rate of 79% (Fig. 4A). The tetrazolium test revealed a higher percentage of non-viable seeds produced by E^+^C^+^ plants (36%) compared with E^+^C^−^ plants (17%). The alkaloid concentration in seeds was negatively correlated with seed germination rate across the treatments (*t* = −5.28, *P* < 0.001, *R*^2^ = 0.65; Fig. 4B). Mycelial biomass in the seeds of E^+^C^−^ and E^+^C^+^ plants was assessed microscopically, and no notable difference in hyphal density was observed (data not shown). Seed germination rate was not correlated with seed biomass (*t* = −0.45, *P* = 0.66, *R*^2^ = −0.05).

### Alkaloid concentrations and aphid numbers of progeny plants

**Fig. 5.**
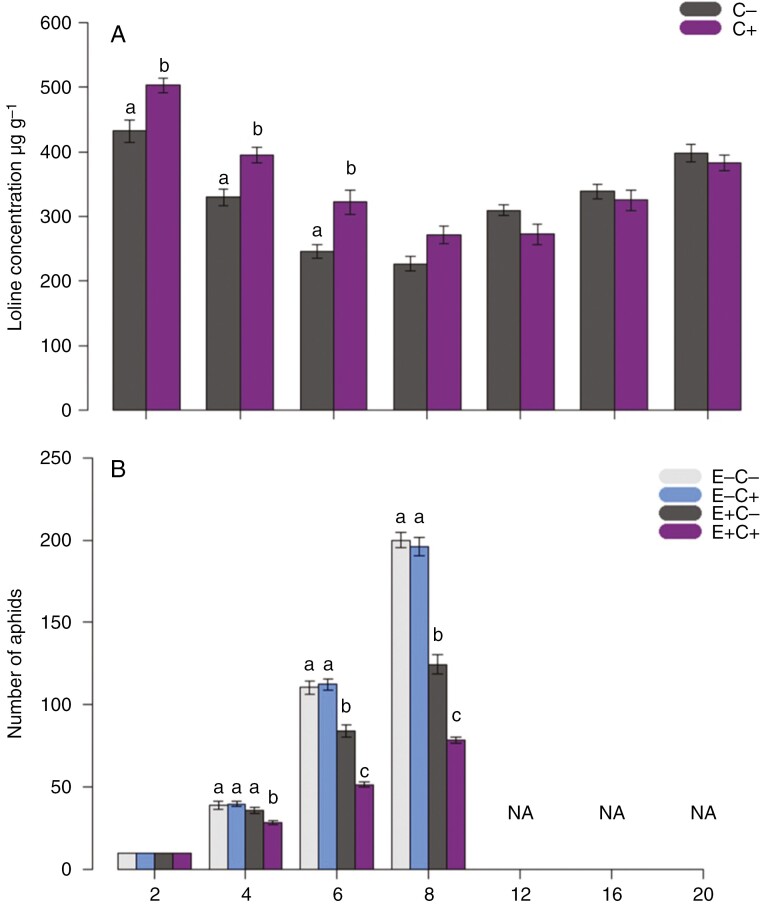
(A) Loline concentration (in micrograms per gram dry weight) in endophyte-symbiotic (E^+^) progeny plants over a period of 20 weeks following seed germination. (B) Number of aphids over a period of 8 weeks after seed germination. Ten aphids were introduced on the seedlings at week 2, and the aphid trial ended at week 8 from the start of the experiment. Treatments in the mother plants were endophyte-symbiotic (E^+^) and endophyte-free (E^−^) in combination with defoliated (C^+^) and non-defoliated (C^−^) plants. Lowercase letters represent results from a generalized linear mixed model accounting for repeated measurements followed by Tukey’s *post hoc* test. Values are presented as the mean ± s.e.; *n* = 40. NA represents non-applicable sampling points when the aphid experiment had ended and loline alkaloid concentrations were measured further.

Alkaloid concentrations of the progeny of E^+^ plants decreased over the first 8 weeks after germination and increased slowly afterwards (Fig. 5A). Progeny of E^+^C^+^ plants had higher loline concentrations 2, 4 and 6 weeks following germination compared with the loline concentrations of E^+^C^−^ progeny (Fig. 5A). At the same time, aphid numbers on progeny of E^+^C^+^ plants increased more slowly in comparison to aphid numbers on E^+^C^−^ plants, resulting in significantly lower aphid numbers on progeny of E^+^C^+^ plants compared with E^+^C^−^ plants after 6 and 8 weeks of aphid feeding (Fig. 5B). Overall alkaloid concentrations were negatively correlated with aphid numbers, demonstrating the defensive attributes of loline alkaloids (Pearson correlation: *t* = −9.67, *P* < 0.01, *r*^2^ = −0.74) (Wilkinson *et al.*, 2000). The difference in alkaloid concentrations between progeny of E^+^C^+^ and E^+^C^−^ disappeared 8 weeks following germination, and alkaloid concentrations increased overall (Fig. 5A).

## DISCUSSION

We recorded higher loline alkaloid concentrations in E^+^C^+^ seeds compared with E^+^C^−^ seeds. High loline alkaloid concentrations were correlated with a reduction in the seed germination rate. In addition, high alkaloid concentrations in seeds caused elevated alkaloid concentrations in progeny of E^+^C^+^ plants compared with progeny of E^+^C^−^ plants during the first 6 weeks of growth, which resulted in better protection against aphid herbivory. The increased investment of alkaloids into seeds was correlated with decreased leaf alkaloid concentrations, which indicates a trade-off in resource allocation between the resistance of the defoliated plant and protection of early ontogenetic stages of its progeny.

Alkaloid biosynthesis is the basis for the defensive mutualistic symbiosis of systemic *Epichloë* endophytes and their cool-season grass hosts and is dependent on nutrient availability (Cheplick *et al.*, 1989; Saikkonen *et al.*, 2004; Schardl *et al.*, 2004). Systemic grass endophytes elicit an elevated photosynthetic activity in their host, indicating their stimulation of nitrogen acquisition, which is particularly needed for alkaloid biosynthesis (Rasmussen *et al.*, 2008; Ryan *et al.*, 2014). Regular removal of large parts of leaf tissue limits photosynthetically active tissue and ultimately limits nitrogen supply, which might limit alkaloid biosynthesis (in contrast to herbivore-induced alkaloid biosynthesis in the studies by Bultman *et al.*, 2004; Rasmussen *et al.*, 2007; Fuchs *et al.*, 2017*b*) In non-endophytic plants, nutrient limitation owing to intense defoliation has been linked to a trade-off between resource allocation to growth/reproduction or resistance (reviewed by Walters and Heil, 2007), indicating a fine-tuned resource allocation to maximize fitness under herbivory. In cases where plant defence is executed by a systemic endophyte, limited nutrient availability can elicit competition between the grass and the endophyte (Müller and Krauss, 2005). The endophyte consumes resources for alkaloid biosynthesis, and these are then lacking from proper seed development of the host grass. In our study, this was indicated by increased alkaloid concentrations and decreased seed viability of seeds of heavily defoliated plants.

Although grass endophytes are often considered plant mutualists, they can become parasitic in low-nutrient conditions, indicating their dominance over resources in competition with the host plant (Faeth and Sullivan, 2003; Müller and Krauss, 2005). We showed an increase in seed alkaloid concentrations despite high defoliation, demonstrating nitrogen investment into defence compounds produced by the endophyte, which was then correlated with lower seed viability (Saikkonen *et al.*, 1998, 1999; Sullivan *et al.*, 2007). This demonstrates how plants can compete over resources with their systemic and, in most situations, symbiotic endophyte, particularly in environments with high herbivore pressure.

Our results demonstrate that the progeny of E^+^C^+^ plants had a defensive advantage over E^+^C^−^ progeny, as seen by reduced aphid population growth. Loline alkaloids are potent insecticides that are highly effective against aphids (Wilkinson *et al.*, 2000; Popay *et al.*, 2023). However, alkaloid concentrations decrease in seedlings/young plants after germination owing to their fast initial growth and a subsequent distribution of seed-stored alkaloids (Fuchs *et al.*, 2017*b*; Hewitt *et al.*, 2020). Therefore, stocking high alkaloid concentrations into seeds has two apparent advantages: (1) protection of seeds from predation; and (2) protection of early ontogenetic stages of progeny plants. Higher alkaloid concentrations in seeds and the subsequent protection of progeny might provide significant survival and competitive advantages in field conditions over seeds with lower alkaloid concentrations (but see Bubica Bustos *et al.*, 2022). High alkaloid concentrations of endophyte-symbiotic plants in areas with high defoliation highlight the evolutionary advantage of investment in alkaloids under high defoliation in natural environments (Francis and Baird, 1989; Bouton *et al.*, 1993; Clay and Schardl, 2002).

Our results further suggest that reproductive strategy might change under high herbivory in endophyte-symbiotic plants from an investment into many viable but less-protected progeny to lower viability of higher-protected progeny (Newell and Tramer, 1978). Considering the high degree of defoliation applied in this experiment, the investment of resources into the protection of the next generation at the cost of seed germination might ultimately be an advantageous evolutionary strategy providing additional protection in an environment with a high herbivore pressure (Sullivan *et al.*, 2007). Future studies need to investigate the mechanisms behind nutrient allocation and trade-offs in endophyte-symbiotic cool-season grasses (Scott *et al.*, 2018). In grasses symbiotic to systemic endophytes, we suggest including the seed stage in studies of the growth–defence trade-off owing to its role in reproduction for both symbiotic partners (Siegel and Bush, 1996; Bush *et al.*, 1997, Bastías *et al.* 2021).

On an eco-evolutionary level, our results provide new possible explanations for the high variability of endophyte frequency found in natural environments (Clay and Schardl, 2002; Faeth and Saikkonen, 2006). For instance, considering a constantly high natural defoliation of a meadow because of grazing, a higher deterrence of herbivores and seed predators via high alkaloid concentrations in seeds might provide a higher survival and fitness advantage for the progeny compared with seeds with lower alkaloid-conferred protection and higher germination rate. In times of lower herbivore pressure, however, the fitness advantage is likely to be shifted to plants producing seeds with lower alkaloid concentrations and higher germination rates (Cheplick and Faeth, 2009), resulting in a mosaic of endophyte-symbiotic and endophyte-free meadows. Although *Epichloë*–grass symbioses commonly invest in high seed sets to increase the fitness of the symbiotum, our results indicate that high defoliation might shift the allocation of resources towards increased protection of the progeny at the expense of seed viability.

## Conclusion

Our study links repeated and severe tissue losses of grass hosting a symbiotic endophyte to an investment of alkaloids into plant seeds at the expense of seed viability, which suggests a potential trade-off in resource allocation between plant and endophyte. The results provide new insights into the nature of the symbiotic relationship between cool-season grasses and systemic *Epichloë* endophytes and might provide a possible explanation for the natural variability of endophyte frequency in natural and semi-natural environments.

## Data Availability

Data are made available upon resonable request.
